# Patterns and correlates of health service contact prior to serious offences by people with severe mental illness

**DOI:** 10.1177/00048674241254213

**Published:** 2024-05-10

**Authors:** Corrie Goodhand, Georgia Lyons, Anina Johnson, Olav Nielssen, Matthew Large, Kimberlie Dean

**Affiliations:** 1 Discipline of Psychiatry and Mental Health, Faculty of Medicine and Health, University of New South Wales, Sydney, NSW, Australia; 2 Department of Psychology, Faculty of Medicine and Health Sciences, Macquarie University, Sydney, NSW, Australia; 3 Justice Health and Forensic Mental Health Network, Matraville, NSW, Australia

**Keywords:** Psychosis, violence, treatment, prevention, prodrome, Forensic Patient

## Abstract

**Background:**

Contact with health services prior to offences committed by people with mental illness is an opportunity for intervention and prevention. This study examines the pattern and correlates of health service contact by people with severe mental illness before a serious offence.

**Method:**

Linkage of a cohort of 477 Forensic Patients found not guilty due to mental illness between 1990 and 2016, and statewide databases of contact with emergency departments, hospital admission and outpatient mental health services in the state of New South Wales, Australia.

**Results:**

A total of 84% of the sample had contact with any health service and 76% had contact with an outpatient mental health service prior to the index offence. About two-thirds of the sample had contact with a mental health service in the year before the offence. Factors independently associated with the absence of contact at any point prior to the index offence were non-English-speaking background, being engaged in employment or study, and an absence of childhood abuse or neglect. Although nearly every Forensic Patient had a psychotic illness at the time of the index offence, psychosis was not diagnosed at the time of 61/106 (57.5%) emergency department presentations, in 54/174 (31.0%) hospital admissions and 149/222 (67.1%) attendances at outpatient mental health services prior to the offence.

**Conclusions:**

Most Forensic Patients had contact with health services prior to their offences but many were not identified as having a psychotic illness. Although the symptoms of psychosis may have emerged in the period between contact and the offence, the findings suggest that emerging or underlying psychosis were missed or attributed to other conditions.

## Introduction

Although most people with severe mental illness will never be violent, there is a well-established association between schizophrenia and violence (Fazel et al., 2009). Symptoms of severe forms of mental illness can contribute to behaviour resulting in serious offences, and most jurisdictions have mechanisms to determine the criminal responsibility of people affected by severe forms of mental illness at the time of their offences. Most high-income countries also have arrangements for the detention and treatment of offenders who are deemed not criminally responsible by reason of mental illness or cognitive impairment, often referred to as ‘Forensic Patients’. The outcomes for released Forensic Patients have been extensively studied (Dean et al., 2021; Fazel et al., 2016), but less attention has been given to opportunities for intervention and prevention before the offence.

The early stages of psychotic illness are a period of increased risk for violence, particularly in first-episode psychosis prior to effective treatment (Humphreys et al., 1992; Nielssen et al., 2012; Winsper et al., 2013). Studies have found evidence of a decline in violent behaviour after the initiation of treatment (Langeveld et al., 2014), suggesting the potential to reduce the incidence of violence through earlier treatment. However, studies examining early intervention services for psychosis have reported mixed results. For example, a Danish randomised controlled trial of assertive early intervention for psychosis did not find a reduction in offending (Stevens et al., 2013), whereas a Canadian non-randomised trial of early intervention for psychosis (Randall et al., 2016) and an American pragmatic randomised clinical trial of specialised early treatment for psychosis (Pollard et al., 2020) reported reduced offending in those who received earlier treatment. Beyond the early stages of psychosis, there is evidence that ongoing engagement with mental health services does reduce criminal justice contact among people with persistent psychotic illness (Hwang et al., 2020), consistent with studies demonstrating low reoffending rates by Forensic Patients released from secure care (Dean et al., 2021; Fazel et al., 2016).

Although the evidence for the effect of specific treatments on the risk of violence for patients with schizophrenia is limited (Quinn and Kolla, 2017), studies linking psychiatric care and offending have shown a lower rate of both minor and serious offending by people with mental illness who are adherent to treatment (Rezansoff et al., 2017; Weatherburn et al., 2021). There is also evidence of reduced violence by those treated with clozapine (Bhavsar et al., 2020; di Giacomo et al., 2020) provided within the framework of involuntary outpatient treatment (Ogilvie and Kisely, 2022). Specifically, treatment with depot antipsychotic medication may prevent relapse, increase treatment compliance and lead to reductions in violence (Fazel et al., 2014; Vasic et al., 2018).

Despite some evidence of a reduction in all forms of violent offences among groups of patients receiving treatment for psychosis, data about the effect of access to early psychosis services and ongoing treatment of schizophrenia on the most serious offences is less encouraging. Studies covering the period after the roll out of specialist early intervention services in Australia, Canada and the United Kingdom, when there was also a marked decline in the overall rate of homicide in each of those jurisdictions, did not find a corresponding decline in the rates of homicide by people with severe mental illness (Flynn et al., 2020; Nielssen and Large, 2022; Penney et al., 2018).

Studies of Forensic Patients have consistently identified a subgroup of patients with very limited or no recorded contact with mental health services prior to their offences. A 2006 New Zealand study of referrals to the Auckland Regional Forensic Mental Health Service found 30.2% had no mental health service contact in the preceding 3 years (Cavney et al., 2012), and a similar Canadian study found that 28.6% had no contact in the previous year (Leclair et al., 2022). However, in each of these studies, more than two-thirds of offenders had more recent health service contacts, and an Australian study found that 45% of homicide offenders with psychosis had contact with health services within the previous month (Nielssen, 2007). The failure of a health contact to prevent offending might be due to a range of factors, including that there were no apparent grounds for involuntary psychiatric treatment in mentally ill patients who refused treatment. There may also have been a failure to recognise emerging psychosis, to diagnose psychosis when it was clearly present, to recognise the severity of the illness and the need for more urgent treatment, the failure to prescribe antipsychotic medication, non-adherence to recommended treatment, and a lack of response to the treatment that was provided (Green et al., 2021).

The aim of the current study was to establish the patterns of contact with health services (defined as emergency department [ED], hospital admissions and ambulatory mental health services) among Forensic Patients and to identify the sociodemographic and clinical characteristics associated with lack of contact or effective intervention.

## Methods

### Sampling and data collection

We examined prior health contacts of a cohort of 477 Forensic Patients found not guilty of a serious offence on the grounds of mental illness (NGMI) according to the existing legislation (*the Crimes Act 1900 and the Mental Health [Forensic Provisions] Act 1990*) in New South Wales (NSW) Australia between 1 January 1990 and 29 July 2016. The cases were identified by records held by the NSW Mental Health Review Tribunal (MHRT), a statutory body set up to make recommendations and decisions about detention, leave and release of all Forensic Patients. Sociodemographic, clinical and criminal justice data were extracted from the NSW MHRT electronic records and paper case files (Dean et al., 2021).

The Centre for Health Record Linkage (CHeReL) linked people in the NSW MHRT files with the NSW Emergency Department Data Collection (EDDC), the NSW Admitted Patient Data Collection and the NSW Mental Health Ambulatory Data Collection datasets using identifying information (including names, aliases, dates of birth and sex) provided by the data custodians. Each person in the cohort was allocated a unique Project-specific Person Number (PPN), and the merged data returned to the study team for analysis.

### Measures

The NSW EDDC contains information on ED presentations at most NSW public hospitals between 1 January 2005 and 31 December 2018. ED diagnoses were classified according to the International Classification of Diseases, 9th Revision, Clinical Modification (ICD-9-CM), International Classification of Diseases, 10th Revision, Australian Modification (ICD-10-AM) or SNOMED CT coding systems. ICD-9-CM and SNOMED CT codes were mapped to the corresponding ICD-10-AM codes. Two categories of diagnosis were examined – the diagnosis made at the time of ED presentations for any reason and presentations with a primary diagnosis of mental or behavioural disorder. Only one diagnosis code per presentation was available for analysis. Mental or behavioural disorders were identified if patients had an ICD-10-AM Chapter V diagnosis (codes F00-F99) recorded in the dataset.

The NSW Admitted Patient Data Collection (APDC) contains records from all NSW public, private, psychiatric and repatriation hospitals in NSW from 1 January 2001 until 31 December 2018. Hospital admissions are recorded as ‘episodes of care’ at the end of a period of stay in hospital by discharge, transfer or death. One principal diagnosis is classified according to the ICD-10-AM. Episodes of care were examined according to any principal diagnosis type and a principal diagnosis with a mental or behavioural disorder.

The NSW Mental Health Ambulatory Data Collection contains information on the assessment, treatment, rehabilitation or care of non-admitted mental health patients from 1 January 2001 until 30 June 2018. This includes mental health day programmes, psychiatric outpatient services and outreach community mental health services. Each activity recorded in the dataset is assigned a unique Service Event ID that links together individual activities performed within a single mental health service contact. The primary diagnosis associated with each contact was classified according to the ICD-10.

### Analysis

Descriptive statistics were generated using data extracted from the MHRT files to provide an overview of the sociodemographic and clinical characteristics of the cohort. The data linkage ascertained the proportion and timing of health contacts. Independent variables included the sociodemographic and clinical sample characteristics and were used to ascertain associations with the dependent variable of not having any mental health–related contacts (ED, inpatient or ambulatory) prior to the index offence using logistic regression and multivariable logistic regression. The same analysis was then conducted to determine associations with having no mental health–related contacts within 1 month prior to the index offence. The dependent variable was chosen to identify potential barriers to accessing mental health services that might act as a protective factor against offending. Analyses were performed using Statistical Package for the Social Sciences (SPSS) version 25.

Patients were only included in the analysis if their index offence was at least 6 months after the start of the relevant health dataset to ensure a window for possible health service contacts prior to the index offence. This resulted in a smaller cohort for each of the different health contacts. Descriptive statistics were generated relating to patients’ health service contacts prior to the index offence, including the number of contacts, timing of the contacts and the principal diagnosis received at these contacts.

### Ethical review

Approval for this study was obtained from the NSW Population and Health Services Research Ethics Committee (AU RED Reference: HREC/18/CIPHS/48; Cancer Institute NSW Reference: 2018HRE1003) and the Aboriginal Health and Medical Research Council (reference no. 1749/20). A waiver of consent was provided by the ethics committee.

## Results

### Sample overview

Table 1 describes the sociodemographic and clinical characteristics of the 307 Forensic Patients included in the final sample. Most were male (258, 84.0%) and were aged between 30 and 60 years old at the time of the index offence (197, 64.2%). Most did not identify as Aboriginal and/or Torres Strait Islander (268, 87.3%) and spoke English as a first language (216, 70.4%). Almost half of the sample (150, 48.9%) had a history of child abuse or neglect. The most common primary diagnoses were schizophrenia related (259, 84.4%), and common co-morbid conditions included substance use disorder (207, 67.4%) and head injury (115, 37.5%).

**Table 1.** Sample characteristics of NGMI forensic patients (*N* = 307).

		*n*	%
Sociodemographic characteristics			
Sex assigned at birth	Female	47	15.3
	Male	258	84.0
Age at index offence	Less than 30 years	94	30.6
	30–60 years	197	64.2
	Over 60 years	16	5.2
Aboriginal and/or Torres Strait Islander	No	268	87.3
	Yes	39	12.7
Language background	English background	216	70.4
	Non-English background	79	25.7
Relationship status	Single/never married	128	41.7
	Married/de facto/divorced	99	32.2
Parental status	No children	178	58.0
	Has children	128	41.7
Education level	Completed year 10	206	67.1
	Below year 10	87	28.3
Any employment or study	No	212	69.1
	Yes	67	21.8
History of child abuse or neglect	No	138	45.0
	Yes	150	48.9
Clinical characteristics			
Primary diagnosis	Schizophrenia related	259	84.4
	Other	47	15.3
Psychopathy diagnosis	No	287	93.5
	Yes	15	4.9
Personality disorder diagnosis	No	259	84.4
	Yes	43	14.0
Substance use disorder diagnosis	No	99	32.2
	Yes	207	67.4
Intellectual disability	No	132	43.0
	Yes	31	10.1
Head injury	No	166	54.1
	Yes	115	37.5
History of self-harm	No	110	35.8
	Yes	179	58.3
First-degree relative with mental illness	No	129	42.0
	Yes	133	43.3

Percentages may not add to 100 due to missing data.

### Any health service contacts

Most of the sample (259, 84.4%) had one or more prior health service contacts (for any diagnosis) in one or more of the three datasets (Table 2). Nearly half (136, 44.3%) had contact within 1 month of the offence and one quarter (75, 24.4%) within 1 week. Three quarters (232, 75.6%) had at least one mental health service contact prior to the index offence (Table 3), and 127 (41.4%) had any mental health contact within a month and 69 (22.5%) within a week. Those who were known to services had a large number of contacts, with the median number of any type of contact per known patient of 37.0 (range: 1–1610; interquartile range [IQR]: 8.0–182.5). The median time between the index offence and the most recent mental health contact was 0.8 months (range: 0.03–111.3; IQR: 0.2–4.3).

**Table 2.** Timing of contacts (any diagnosis) occurring prior to the index offence (maximum follow-up period of 14.33 years).

Timing of most recent contact prior to the index offence	Emergency department data collection (*N* = 235)		Admitted patient data collection (*N* = 307)		Mental health ambulatory data collection (*N* = 307)		Any health contact (*N* = 307)	
	*n* (%)	Cumulative *n* (%)	*n* (%)	Cumulative *n* (%)	*n* (%)	Cumulative *n* (%)	*n* (%)	Cumulative *n* (%)
1 week or less	15 (6.4)	15 (6.4)	7 (2.3)	7 (2.3)	66 (21.5)	66 (21.5)	75 (24.4)	75 (24.4)
1–2 weeks	10 (4.3)	25 (10.6)	6 (2.0)	13 (4.2)	21 (6.8)	87 (28.3)	25 (8.1)	100 (32.6)
2 weeks–1 month	8 (13.4)	33 (14.0)	12 (3.9)	25 (8.1)	34 (11.1)	121 (39.4)	36 (11.7)	136 (44.3)
1–2 months	18 (7.7)	51 (21.7)	28 (9.1)	53 (17.3)	21 (6.8)	142 (46.3)	25 (8.1)	161 (52.4)
2–6 months	34 (14.5)	85 (36.2)	39 (12.7)	92 (30.0)	31 (10.1)	173 (56.4)	41 (13.4)	202 (65.8)
6–12 months	29 (12.3)	114 (48.5)	45 (14.7)	137 (44.6)	23 (7.5)	196 (63.8)	23 (7.5)	225 (73.3)
More than 12 months	63 (26.8)	177 (75.3)	86 (28.0)	223 (72.6)	26 (8.5)	222 (72.3)	34 (11.1)	259 (84.4)
No contact recorded	58 (24.7)	235 (100.0)	84 (27.4)	307 (100.0)	85 (27.7)	307 (100.0)	48 (15.6)	307 (100.0)

**Table 3.** Timing of health contacts (primary diagnosis of mental or behavioural disorder) occurring prior to the index offence (maximum follow-up period of 14.33 years).

Timing of most recent contact prior to the index offence	Emergency department data collection (*N* = 235)		Admitted patient data collection (*N* = 307)		Mental health ambulatory data collection (*N* = 307)		Any mental health contact (*N* = 307)	
	*n* (%)	Cumulative %	*n* (%)	Cumulative %	*n* (%)	Cumulative %	*n* (%)	Cumulative %
1 week or less	5 (2.1)	5 (2.1)	<5	<5	66 (21.5)	66 (21.5)	69 (22.5)	69 (22.5)
1–2 weeks	6 (2.6)	11 (4.7)	5 (1.6)	7 (2.3)	21 (6.8)	87 (28.3)	22 (7.2)	91 (29.6)
2 weeks–1 month	5 (2.1)	16 (6.8)	11 (3.6)	18 (5.9)	34 (11.1)	121 (39.4)	36 (11.7)	127 (41.4)
1–2 months	12 (5.1)	28 (11.9)	20 (6.5)	38 (12.4)	21 (6.8)	142 (46.3)	23 (7.5)	150 (48.9)
2–6 months	19 (8.1)	47 (20.0)	32 (10.4)	70 (22.8)	31 (10.1)	173 (56.4)	33 (10.7)	183 (59.6)
6–12 months	16 (6.8)	63 (26.8)	33 (10.7)	103 (33.6)	23 (7.5)	196 (63.8)	20 (6.5)	203 (66.1)
More than 12 months	43 (18.3)	106 (45.1)	71 (23.1)	174 (56.7)	26 (8.5)	222 (72.3)	29 (9.4)	232 (75.6)
No contact recorded	129 (54.9)	235 (100.0)	133 (43.3)	307 (100.0)	85 (27.7)	307 (100.0)	75 (24.4)	307 (100.0)

### Emergency department presentations

For the 235 whose offence took place at least 6 months after the start of EDDC data availability, 177 (75.3%) had at least one ED presentation. The median number of pre-index offence ED presentations per patient was 4.0 (range: 1–150; IQR: 2–10) and the median time between the index offence and most recent ED presentation was 7.0 months (range: 0.03–81.2; IQR: 1.6–18.3). About half (114, 48.5%) had their most recent ED presentation within 12 months of the index offence, 33 (14.0%) within 1 month and 15 (6.4%) in the week before the index offence. A diagnosis of a mental health or behavioural disorder was recorded in about a third of those presenting to ED (62, 35.0%), and just under one quarter had diagnoses related to injuries, poisoning and intentional self-harm (40, 22.6%) (Table 4).

**Table 4.** Principal diagnosis received at the most recent hospital contact for any reason recorded as occurring prior to the index offence.

Diagnostic category	Emergency department (*N* = 177)	Hospital admission (*N* = 223)
	*n* (%)	*n* (%)
Mental and behavioural disorders	62 (35.0)	139 (62.3)
Injuries and poisoning, including intentional self-harm	40 (22.6)	20 (9.0)
Other symptoms not elsewhere classified	23 (13.0)	8 (3.6)
Other specific diseases^a^	16 (9.0)	24 (10.8)
Other factors influencing health status	9 (5.1)	6 (2.7)
Diseases of the digestive system	6 (3.4)	11 (4.9)
Pregnancy, childbirth, puerperium and the perinatal period	<5	7 (3.1)
Infectious and parasitic diseases	<5	<5
Neoplasms	-	<5
Diseases of the skin and subcutaneous tissue	-	<5
Unknown	20 (11.3)	< 5

a Diseases of the musculoskeletal system, nervous system, eye/adnexa, circulatory system, respiratory system and genitourinary system.

Nearly half (106, 45.1%) had at least one mental health–related ED presentation recorded as occurring prior to their index offence date. The median number of mental health–related ED presentations per patient was 2.0 (range: 1–51; IQR: 1.0–5.25), and the median time between the most recent mental health presentation and the index offence was 7.8 months (range: 0.03–97.9; IQR: 1.9–18.4). Around a quarter (63, 26.8%) had a mental health presentation within 12 months of the index offence date. Of those patients who presented to the ED for mental health reasons, 46 (43.4%) were diagnosed with schizophrenia spectrum disorders, with neurotic, stress-related and somatoform disorders being the next most common diagnostic category (19, 17.9%) (Table 5).

**Table 5.** Principal diagnosis received at the most recent health contact for mental health reasons recorded as occurring prior to the index offence.

Diagnostic category	Emergency department (*N* = 106)	Hospital admission (*N* = 174)	Ambulatory mental health (*N* = 222)
	*n* (%)	*n* (%)	*n* (%)
Schizophrenia, schizotypal and delusional disorders	46 (43.4)	120 (69.0)	73 (32.9)
Neurotic, stress-related and somatoform disorders	19 (17.9)	<5	<5
Mood disorders	12 (11.3)	15 (8.6)	11 (5.0)
Disorders of adult personality and behaviour	8 (7.5)	7 (4.0)	<5
Disorders with onset occurring in childhood/adolescence	8 (7.5)	<5	<5
Disorders due to psychoactive substance use	5 (4.7)	24 (13.8)	12 (5.4)
Other,^a^ including unspecified mental disorder	8 (7.5)	<5	17 (7.7)
Diagnosis not yet allocated	-	-	74 (33.3)
Unknown	-	-	29 (13.1)

a Organic and symptomatic mental disorders, Behavioural syndromes associated with physiological disturbances and Disorders of psychological development.

### Hospital admissions

Of the 307 whose index offence took place at least 6 months after the start of APDC data availability, 223 (72.6%) had at least one hospital admission. The median number of pre-offence hospital admissions was 5.0 (range: 1–120; IQR: 2.0–10.0), and the median time between the index offence and most recent admission was 8.3 months (range: 0.03–116.1; IQR: 2.3–22.0). Again, a little under half (137, 44.6%) had their most recent hospital admission within 12 months of the index offence, 25 (8.1%) within 1 month and 7 (2.3%) within 1 week, including several whose offences took place in hospital. The median duration of the most recent admission was a relatively brief 5.0 days (range: 1–593; IQR: 1.0–16.0). A significant number (19, 8.5%) were coded as a ‘Justice Health’ admission, indicating the individual was receiving treatment in a prison mental health unit. However, only around two-thirds (139, 62.3%) had a principal diagnosis of mental or behavioural disorder at the time of their most recent admission, with the second most common diagnostic category being injuries, poisoning or intentional self-harm (20, 9.0%) (Table 4).

More than half (174, 56.7%) had at least one pre-index offence hospital admission with a principal diagnosis of mental or behavioural disorder. The median number of admissions per patient was 4.0 (range: 1–73; IQR: 2.0–8.0), the median length of admission was 10.0 days (range: 1–593; IQR: 2.0–25.25) and 81 (46.6%) of those had an involuntary stay in a psychiatric unit. Around one-third of the cohort (103, 33.6%) had their most recent mental health–related admission within 12 months of the index offence. For all those who had been admitted, the median time between the index offence and the most recent mental health–related hospital admission was 8.5 months (range: 0–98.0; IQR: 2.5–21.4). The most common diagnoses at the time of admission were schizophrenia spectrum disorders (69.0%; *n* = 120) and disorders due to psychoactive substance use (13.8%; *n* = 24) (Table 5).

### Ambulatory mental health contacts

Of the 307 whose index offence took place at least 6 months after the start of the ambulatory mental health data availability, 222 (72.3%) had at least one contact recorded prior to their index offence, and the median number of contacts was 39.0 (range: 1–1592; IQR: 9.8–185.3). The median duration between the most recent ambulatory mental health contact and the index offence was 0.8 months (25 days) (range: 0–111.3; IQR: 0.2–4.6). Two hundred twenty-five (73.3%) had a mental health service contact within 12 months of the index offence and *n* = 66 (21.6%) had a contact within 1 week. A third (74, 33.3%) did not have a diagnosis recorded at the most recent contact, and another third (73, 32.9%) had a primary diagnosis of schizophrenia spectrum disorders at their most recent contact, compared to 84.4% who had a primary diagnosis of schizophrenia recorded in the MHRT files following the index offence.

### Predictors of having no mental health contacts prior to the index offence

Patients were less likely to have a mental health–related contact prior to their index offence if they were from a non-Aboriginal or Torres Strait Islander background (odds ratio [OR] = 4.4; 95% confidence interval [CI] = [1.3, 14.8]), non-English-speaking background (OR = 3.4; 95% CI = [2.0, 6.0]), were engaged in employment or study (OR = 5.4; 95% CI = [3.0, 9.9]) and had no history of child abuse or neglect (OR = 2.1; 95% CI = [1.2, 3.7]). Patients were less likely to have a mental health–related contact if they did not have a co-morbid substance use disorder (OR = 1.8; 95% CI = [1.1, 3.1]) and did not have a first-degree relative with a mental illness (OR = 1.8; 95% CI = [1.0, 3.2]) (Table 6).

**Table 6.** Bivariate and multivariable logistic regression analysis for no mental health–related contacts prior to the index offence.

		No contact (*N* = 75)	Any contact (*N* = 232)	Odds ratios [95% CI]	Adjusted odds ratio [95% CI]
		*n* (%)	*n* (%)		
Sociodemographic characteristics					
Sex assigned at birth	Female	9 (12.0)	38 (16.4)	1.0 (reference)	-
	Male	65 (86.7)	193 (83.2)	1.4 [0.7, 3.1]	
Age at index offence	Less than 30 years	22 (29.3)	72 (31.0)	1.0 (reference)	-
	30–60 years	47 (62.7)	150 (64.7)	1.9 [0.7, 5.6]	
	Over 60 years	6 (8.0)	10 (4.3)	2.0 [0.6, 6.0]	
Aboriginal and/or Torres Strait Islander	Yes	<5	36 (15.5)	**1.0 (reference)**	1.0 (reference)
	No	72 (96.0)	196 (84.5)	4.4 [1.3, 14.8]*	1.6 [0.4, 6.0]
Language background	English background	39 (52.0)	177 (76.3)	**1.0 (reference)**	**1.0 (reference)**
	Non-English background	34 (45.3)	45 (19.4)	3.4 [2.0, 6.0]***	3.1 [1.4, 6.6]**
Relationship status	Single/never married	24 (32.0)	104 (44.8)	1.0 (reference)	-
	Married/de facto/divorced	29 (38.7)	70 (30.2)	1.8 [1.0, 3.3]	
Parental status	No children	42 (56.0)	136 (58.6)	1.0 (reference)	-
	Has children	32 (42.7)	96 (41.4)	1.1 [0.6, 1.8]	
Education level	Completed year 10	48 (64.0)	158 (68.1)	1.0 (reference)	-
	Below year 10	21 (28.0)	66 (28.4)	1.1 [0.6, 1.9]	
Any employment or study	No	34 (45.3)	178 (76.7)	**1.0 (reference)**	**1.0 (reference)**
	Yes	34 (45.3)	33 (14.2)	5.4 [3.0, 9.9]***	4.0 [1.9, 8.1]***
History of child abuse or neglect	Yes	27 (36.0)	123 (53.0)	**1.0 (reference)**	**1.0 (reference)**
	No	44 (58.7)	94 (40.5)	2.1 [1.2, 3.7]**	2.1 [1.0, 4.1]*
Clinical characteristics					
Primary diagnosis	Schizophrenia related	59 (78.7)	200 (86.2)	1.0 (reference)	-
	Other	16 (21.3)	31 (13.4)	1.8 [0.9, 3.4]	
Psychopathy diagnosis	No	70 (93.3)	217 (93.5)	1.0 (reference)	-
	Yes	<5	11 (4.7)	1.2 [0.4, 3.7]	
Personality disorder diagnosis	Yes	10 (13.3)	33 (14.2)	1.0 (reference)	-
	No	64 (85.3)	195 (84.1)	1.1 [0.5, 2.3]	
Substance use disorder diagnosis	Yes	43 (57.3)	164 (70.7)	**1.0 (reference)**	1.0 (reference)
	No	32 (42.7)	67 (28.9)	1.8 [1.1, 3.1]*	1.3 [0.6, 2.6]
Intellectual disability	Yes	<5	27 (11.6)	1.0 (reference)	-
	No	39 (52.0)	93 (40.1)	2.8 [0.9, 8.6]	
Head injury	Yes	25 (33.3)	90 (38.8)	1.0 (reference)	-
	No	46 (61.3)	120 (51.7)	1.4 [0.8, 2.4]	
History of self-harm	Yes	35 (46.7)	144 (62.1)	1.0 (reference)	-
	No	32 (42.7)	78 (33.6)	1.7 [1.0, 2.9]	
First-degree relative with mental illness	Yes	26 (34.7)	107 (46.1)	**1.0 (reference)**	1.0 (reference)
	No	39 (52.0)	90 (38.8)	1.8 [1.0, 3.2]*	1.0 [0.5, 2.1]

CI: confidence interval.

* *p* < 0.05; **p < 0.01; ***p < 0.001.

Adjusted odds ratios (AORs) calculated using multivariable logistic regression suggested non-English-speaking background (AOR = 3.1; 95% CI = [1.4, 6.6]), being engaged in employment or study (AOR = 4.0; 95% CI = [1.9, 8.1]) and not having a history of child abuse or neglect (AOR = 2.1; 95% CI = [1.0, 4.1]) were associated with a lack of health contacts, while Aboriginal and Torres Strait Islander background, substance use disorder and family history of mental illness were not significantly associated with not having a mental health–related contact prior to the index offence (Table 6).

Patients were significantly less likely to have a contact within 1 month if they were from a non-English-speaking background (OR = 1.9; 95% CI = [1.1, 3.2]) and were engaged in any employment or study (OR = 3.7; 95% CI = [1.9, 7.0]). Being engaged in employment or study also remained significant at the multivariable level (AOR = 3.5; 95% CI = [1.8, 6.7]) (Table 7).

**Table 7.** Bivariate and multivariable logistic regression analysis for no mental health–related contact within 1 month of the index offence.

		No contact (*N* = 180)	Any contact (*N* = 127)	Odds ratios [95% CI]	Adjusted odds ratio [95% CI]
		*n* (%)	*n* (%)		
Sociodemographic characteristics					
Sex assigned at birth	Female	25 (13.9)	22 (17.3)	1.0 (reference)	-
	Male	153 (85.0)	105 (82.7)	1.3 [0.7, 2.4]	
Age at index offence	Less than 30 years	50 (27.8)	44 (34.6)	1.00 (reference)	-
	30–60 years	119 (66.1)	78 (61.4)	1.3 [0.8, 2.2]	
	Over 60 years	11 (6.1)	5 (3.9)	1.9 [0.6, 6.0]	
Aboriginal and Torres Strait Islander background	Yes	19 (10.6)	20 (15.7)	1.0 (reference)	
	No	161 (89.4)	107 (84.3)	1.6 [0.8, 3.1]	
Language background	English background	116 (64.4)	100 (78.7)	**1.0 (reference)**	1.0 (reference)
	Non-English background	54 (30.0)	25 (19.7)	1.9 [1.1, 3.2]*	1.6 [0.9, 2.8]
Relationship status	Single/never married	63 (35.0)	65 (51.2)	1.0 (reference)	-
	Married/de facto/divorced	59 (32.8)	40 (31.5)	1.2 [0.9, 2.6]	
Parental status	No children	100 (55.6)	78 (61.4)	1.0 (reference)	-
	Has children	79 (43.9)	49 (38.6)	1.3 [0.8, 2.0]	
Education level	Completed year 10	119 (66.1)	87 (68.5)	1.0 (reference)	-
	Below year 10	51 (28.3)	36 (28.3)	1.0 [0.6, 1.7]	
Any employment or study	No	108 (60.0)	104 (81.9)	**1.0 (reference)**	**1.0 (reference)**
	Yes	53 (29.4)	14 (11.0)	3.7 [1.9, 7.0]***	3.5 [1.8, 6.7]***
History of child abuse or neglect	Yes	81 (45.0)	69 (54.3)	1.0 (reference)	**-**
	No	87 (48.3)	51 (40.2)	1.5 [0.9, 2.3]	
Clinical characteristics					
Primary diagnosis	Schizophrenia related	32 (17.8)	15 (11.8)	1.0 (reference)	-
	Other	148 (82.2)	111 (87.4)	1.6 [0.8, 3.1]	
Psychopathy diagnosis	No	168 (93.3)	119 (93.7)	1.0 (reference)	-
	Yes	9 (5.0)	6 (4.7)	1.1 [0.4, 3.1]	
Personality disorder diagnosis	Yes	151 (83.9)	108 (85.0)	1.0 (reference)	-
	No	26 (14.4)	17 (13.4)	1.1 [0.6, 2.1]	
Substance use disorder diagnosis	Yes	119 (66.1)	88 (69.3)	1.0 (reference)	-
	No	60 (33.3)	39 (30.7)	1.1 [0.7, 1.9]	
Intellectual disability	Yes	19 (10.6)	12 (9.4)	1.0 (reference)	-
	No	82 (45.6)	50 (39.4)	1.0 [0.5, 2.3]	
Head injury	Yes	93 (51.7)	73 (57.5)	1.0 (reference)	-
	No	69 (38.3)	46 (36.2)	1.2 [0.7, 1.9]	
History of self-harm	Yes	98 (54.4)	81 (63.8)	1.0 (reference)	-
	No	70 (38.9)	40 (31.5)	1.5 [0.9, 2.4]	
First-degree relative with mental illness	Yes	74 (41.1)	59 (46.5)	1.0 (reference)	-
	No	82 (45.6)	47 (37.0)	1.4 [0.9, 2.3]	

CI: confidence interval.

* *p* < 0.05; **p < 0.01; ***p < 0.001.

## Discussion

The main findings of the study were that more than four-fifths of the cohort of Forensic Patients had contact with ED, hospitals or ambulatory mental health care on at least one occasion prior to the index offence. Three quarters of the cohort had one or more mental health–related contacts, and two-fifths had contact in the month before the offence. These high rates of contact suggest that some serious violent offences might have been avoided if psychotic illness had been identified and adequately treated. Almost all of the cohorts were later diagnosed with a psychotic illness, but many were not recorded as having this type of illness at their most recent health contact, including during psychiatric hospital admission.

The study also showed a significant proportion had not had any secondary health service contact prior to the offence. Patients may have been more likely to have prior contact if they had pre-existing difficulties and exposure to psychiatric care, or were better able to access services. However, the contact they did have may not have been during the episode of illness in which the offence took place, and prior diagnoses might have contributed to the failure to recognise the emergence of psychotic illness.

The proportion of Forensic Patients with a documented history of at least some health service contact, including for a mental condition, is comparable to the results of studies from New Zealand and Canada, in which around 70% had prior contact with mental health services (Cavney et al., 2012; Leclair et al., 2022). These studies also identified a significant proportion of serious offenders with severe mental illness who had no prior contact with mental health services, a number of whom had long durations of untreated psychosis (Large et al., 2008). Studies of the duration of untreated psychosis have identified multiple barriers to care, including lack of understanding the psychotic symptoms by the affected person and their relatives and lack of awareness of services that are available (Nielssen et al., 2012). The finding of this study suggests that being from a non-English-speaking background is a further barrier to care, either due to cultural interpretations of symptoms and nature and treatment of mental illness, reduced awareness of available services and difficulties communicating with clinicians.

There was a wide variation in the duration between the last contact with services and the index offence. Most of the mental health service contact in the month prior to the index offence was with ambulatory mental health services, suggesting they were either receiving treatment, or that services were attempting to arrange treatment, at the time of the index offence. Contact with an ED for a mental health reason may have indicated the recent onset of illness, emergence of acute symptoms in a person known to have mental illness, poor engagement or difficulty obtaining access to other mental health care. However, only a quarter of the eligible sample attended an ED for a mental health condition in the year before the offence, and only 6.8% in the month before of the index offence, which was a higher proportion than a Queensland study of Forensic Patients, which found only 8.9% had contact with an ED in the 12 months prior to the index offence (Green et al., 2021). That study reported a number of potentially negative treatment events, including discharge from a service, ending of a period of involuntary treatment and change in diagnosis, which showed that the fact of contact alone did not necessarily mean an intervention took place or that treatment was initiated. However, the relatively small proportion of both studies with recent ED contact suggests that EDs have a relatively small role to play in prevention of serious violence by people with psychotic illness.

Although the current study could not identify the specific nature of contact or the outcome, it was notable that the diagnosis recorded at the time of health contact was often for disorders other than psychotic illness, including neurotic disorders, substance abuse and personality disorder. Mood disorders, substance use disorder and maladaptive personality traits are common co-morbid conditions in Forensic Patients (Dean et al., 2021), but the failure to identify a psychotic illness may have been due to the failure to elicit symptoms of underlying psychosis or a pejorative attribution to substance abuse and self-defeating behaviour.

When any type of health service contact was considered, the most common primary physical health problem recorded was an injury or poisoning, including intentional self-harm, confirming the very high level of distress experienced by people with psychotic illness. Self-harm is well known to be associated with psychosis (Harvey et al., 2008), including among Forensic Patients (Justice Health and Forensic Mental Health Network, 2018), and a high rate of untreated psychosis is reported among survivors of serious forms of self-harm (Nielssen et al., 2010). Presentations to health services following self-harm in the context of psychosis may indicate an increased risk of subsequent violence (Witt et al., 2013), as well as increased future risk of further self-harm and suicide.

Multivariable analysis found that Forensic Patients were less likely to have a mental health–related contact at any point prior to the index offence if they were from a non-English-speaking background, were engaged in employment or study, or had no history of childhood maltreatment. Those engaged in employment or education were also identified on multivariable analysis when the outcome was defined as having mental health contact within a month of the index offence. Beyond these few factors, the sociodemographic and clinical characteristics of the two groups were similar. The findings with regard to non-English-speaking Forensic Patients are consistent with a German study that found a greater risk of homicide during psychosis among non-German-speaking migrants (Erb et al., 2001) and indicate a need for measures to improve access to services for people from culturally and linguistically diverse backgrounds. This study also found that people who were employed or engaged in study and those who were not adversely affected by child abuse were less likely to have prior health contacts, which might be because of a greater functional capacity and lower levels of general distress, or the absence of contact because of other conditions, given the number who were not known to have psychosis.

### Strengths and limitations

The strengths of the current study include the use of a total population cohort, the number of variables examined and linkage with multiple statewide administrative data sources. The latter in particular minimises the information (i.e. recall and reporting) and sampling biases (i.e. selection and attrition). In contrast to the few previous studies examining health service contacts before offences, the current study was able to examine contact with different types of health services separately and to extract the diagnoses made at the time of that contact. However, the limitations include the lack of certain types of data, including detailed information about the nature of health service contact, the outcome and types of treatment received, and level of compliance with medication. A separate limitation is the absence of information about federally subsidised private health services including with primary care and private psychiatrists and psychologists, secondary health care delivered outside of NSW and care provided by other non-health or non-government agencies.

A further limitation flows from the sources of health care data. The data collected, including the reasons for the health contact, were recorded in the course of routine health care and were not collected for the purposes of research. This limited the range of independent variables for any possible analyses and may have led to data being missed or coded incorrectly. The sample size, although large and recruited over a long period, was not large enough to show differences in many variables. Finally, the study was not able to examine health service contacts by the much larger group of people who live with psychosis in the community without causing harm to others, as it is quite possible that any analyses of their health contacts would have similar results.

## Conclusion

The results confirm the need for earlier recognition and intervention to treat severe forms of psychotic illness, especially never treated psychosis, even if the person has been unwell for some time. Systems to improve the continuity of care in people known to have persisting psychotic illness might prevent some acts of serious violence and might even lower the overall rate of serious violence and other offending by people with mental illness. Routinely collected health data should also be used effectively to monitor for trends in rates of violence for those with psychosis over time and across jurisdictions. Despite the increased risk of both serious violence and self-harm, the base rates are still too low to identify individuals at particular risk, and the alternative must be to provide an adequate standard of assessment, intervention and follow-up to all patients, and to reduce barriers to care. The results of this study support the needs for systems of care that can provide assertive intervention for emerging psychotic illness and consistent ongoing care for people with persistent psychosis.
